# Protective effect of Idelalisib on carbon tetrachloride‐induced liver fibrosis via microRNA‐124‐3P/phosphatidylinositol‐3‐hydroxykinase signalling pathway

**DOI:** 10.1111/jcmm.17039

**Published:** 2021-11-07

**Authors:** Xiaohe Li, Hailong Li, Shanshan Zhang, Ruotong Zhang, Jinhe Li, Yiying Wei, Cheng Yang, Fubo Zhang, Honggang Zhou

**Affiliations:** ^1^ The State Key Laboratory of Medicinal Chemical Biology College of Pharmacy and Key Laboratory of Molecular Drug Research Nankai University Tianjin China; ^2^ High‐throughput Molecular Drug Screening Centre Tianjin International Joint Academy of Biomedicine Tianjin China; ^3^ Organ Transplantation Center Tianjin First Central Hospital Tianjin China

**Keywords:** hepatic stellate cells, Idelalisib, liver fibrosis, miR‐124‐3p, PI3K/Akt/FOXO3

## Abstract

Liver fibrosis is the repair process of abnormal connective tissue hyperplasia after liver damage caused by different causes. Inhibition of PI3K/Akt signalling pathway can reduce the deposition of extracellular matrix, inhibit the proliferation of hepatic stellate cells (HSCs), and promote its apoptosis to achieve the purpose of therapy. This study aimed to investigate the effect of Idelalisib (PI3K inhibitor) on carbon tetrachloride (CCl_4_)‐induced liver fibrosis in mice. We used CCl_4_‐induced liver fibrosis mouse model in vivo and TGF‐β1‐stimulated HSCs to evaluate the antifibrosis activity of Idelalisib. In vivo, Idelalisib significantly alleviated CCl_4_‐induced liver damage, collagen deposition, and hydroxyproline accumulation in mice. Immunohistochemistry and Western blot results showed that Idelalisib could significantly inhibit the expressions of COL1 and α‐SMA in a concentration‐dependent manner. In cell experiments, Idelalisib significantly inhibited the expressions of COL1, SMA, and p‐Smad3 in TGF‐β‐induced HSCs, thereby inhibiting HSC activation. Flow cytometry and Western blot results showed that Idelalisib significantly promoted TGFβ‐induced apoptosis of HSCs after 48 h of administration, but had no significant effect after 24 h. Idelalisib promoted the apoptosis of activated HSCs by inhibiting the PI3K/Akt/FOXO3 signalling pathway. To further explore the mechanism by which Idelalisib inhibited PI3K, we predicted the miRNA targeting PI3K through the database and crossed it with the down‐regulated miRNA reported in liver fibrosis mice in the past five years. Finally, we identified miR‐124‐3p and miR‐143‐3p. We then demonstrated that Idelalisib significantly promoted miR‐124‐3p and miR‐142‐3p in vitro and in vivo. Dual‐luciferase report analysis showed that Idelalisib significantly inhibited luciferase activity but had no significant effect on the luc‐MUT transfection assay. Finally, we demonstrated that Idelalisib reversed the effects of miR‐124‐3p inhibitor on the PI3K/Akt/FOXO3 asterisk pathway and caspase‐3. Idelalisib has potential as a candidate drug for alleviating liver fibrosis.

## 1 INTRODUCTION

Liver fibrosis refers to the dynamic process of excessive accumulation of extra‐cellular matrix (ECM) in the liver; it is caused by various chronic pathogenic factors, such as hepatitis virus, alcohol, the abnormal function of the metabolic system, chemical drugs, and toxicosis.^1^ In recent years, in‐depth research on the cellular and molecular mechanisms in liver fibrosis development revealed that the development of liver fibrosis is progressive; if the intervention and treatment are carried out in the early stage, then the process of liver fibrosis can be effectively prevented or even reversed.^2^, ^3^ Studies on animal models of liver fibrosis have found that chronic liver injury caused by various factors eventually activated HSCs; thus, the activation of HSCs is among the important markers of liver fibrosis.^4^ The development of a candidate drug that can effectively inhibit the activation of HSCs is of great importance for the clinical reversal of liver fibrosis.

The PI3K/Akt pathway is among the most important pathways discovered; it is widely involved in cell physiological activities and plays a significant regulatory role in cell apoptosis, growth, differentiation, protein translation, and other diseases.^5^ Studies have shown that specific knockout of PI3KR1 in HSCs significantly reduced the expression of extracellular matrix proteins. In addition, the apoptosis of activated HSCs significantly increased.^6^ Mi et al.^7^ showed that maltitol promoted the apoptosis of activated HSCs by inhibiting PI3K/ Akt signalling pathway, thus delaying the progression of pulmonary fibrosis in mice. Therefore, the inhibition of PI3K/ Akt signalling pathway can effectively inhibit liver fibrosis progression. Idelalisib is the world's first highly selective oral phosphatidylinositol 3‐kinase δ (PI3K‐δ) inhibitor that is used to treat relapsed chronic lymphocytic leukaemia, relapsed follicular lymphoma, relapsed small lymphocytic lymphoma, and other malignant tumours; it has good clinical efficacy, safety, and tolerability.^8^, ^9^ However, its role in liver fibrosis remains unclear. Based on previous studies, Idelalisib may effectively inhibit HSC activation and reverse the progression of liver fibrosis.

Further research on the mechanism of liver fibrosis showed that the abnormal expression of miRNA may be related to hepatic fibrosis.^10^, ^11^ The overexpression of miR‐194 inhibits targeted protein kinases to reduce α‐SMA and COL1 protein expressions, blocks cell cycle, and inhibits the activation and proliferation of HSCs, thereby alleviating liver fibrosis.^12^ MicroRNA‐124 (miR‐124) is widely expressed in human and animal organs and is closely related to the pathogenesis of many diseases. Initial research showed that it plays an important role in neurological diseases, including Alzheimer's disease, Parkinson's disease, and ischemic stroke.^13^ Over time, researchers found that miR‐124 is also involved in the pathogenesis of cardiovascular disease, fibrosis, hypertension, and atherosclerosis.^14^ In addition, it is abnormally expressed in many cancers. Interestingly, miR‐143‐3p interacts directly with PI3K, and miR‐124‐3p can be upregulated by rosiglitazone in activated hepatic stellate cells, thereby alleviating liver fibrosis.^15^, ^16^ The miR‐124‐3p/PI3K signalling pathway may be an important pathway to alleviate liver fibrosis.

This study aimed to verify the effect of Idelalisib on CCl_4_‐induced hepatic fibrosis in mice and on the inhibition of TGF‐β‐induced HSCs cell activation. We also examined whether Idelalisib alleviates liver fibrosis by inhibiting PI3K expression through miR‐124‐3p to inhibit the activation of hepatic stellate cells and to promote the apoptosis of activated HSCs.

## 2 MATERIALS AND METHODS

### 2.1 Material

Idelalisib (with a purity of >95%) was purchased from Meilun Biotechnology Co., Ltd. Masson stain, DMEM medium, and PBS buffer powder packs were purchased from Solibol. Apoptosis Assay Kit, Luciferase Assay Kit, and BCA Assay Kit were purchased from Beyotime. Associated antibody, GAPDH, caspase‐9, and cleaved caspase‐9 were purchased from Proteintech Group, Inc. α‐SMA, COL1, PARP, Tublin, P‐PI3K, and PI3K were purchased from Affinity Biosciences. P‐Smad3, Smad3, cleaved caspase‐3, caspase‐3, p‐Akt, and Akt were purchased from Cell Signaling Technology. LX2 cells and LO2 cells were purchased from the ATCC cell bank. Luciferase plasmids, miRNA mimics, and miRNA inhibitors were purchased from Riebok Biotech, Inc.

### 2.2 Animal models

C57BL/6 male mice weighing approximately 18–22 g and with ages of 6–8 weeks old were purchased from Weitong Lihua Co., Ltd. Mice were randomly divided into four groups, namely normal group, CCl_4_ model group, CCl_4_+ Idelalisib (30 mg/kg), and CCl_4_+ Idelalisib (60 mg/kg). Each group included 10 mice. Before the model was established, the body weight of mice was recorded. From 1 week to 4 weeks, mice in the normal group were given corresponding doses of olive oil by intraperitoneal injection, whereas the other groups were given 20% CCl_4_ solution (soluble in olive oil) with 100 μl each. From 5 weeks to 8 weeks, the normal and CCl_4_ groups were given intragastric administration of solvent, whereas the Idelalisib treatment group was given 30 and 60 mg/kg. The mice were weighed, and their weights were recorded every Saturday for 8 weeks. Finally, the liver tissue of mice was obtained, and the morphology of liver tissue was observed. All animal care and laboratory procedures were approved by the Animal Care and Use Committee of Nankai University (IACUC) (License No. SYXK 2019‐0001).

### 2.3 Hematoxylin‐eosin staining

The formaldehyde‐fixed liver tissue was placed into a marked embedding box, rinsed with flowing water for 4 h, and then placed into a tissue dehydrator to dehydrate. The tissue was embedded and sliced into 0.45 μm slices. Then, hematoxylin‐eosin (HE) staining was performed according to the experimental steps. Finally, the tissue was observed under a positive microscope.

### 2.4 Masson staining

According to Masson staining instructions, Weigert iron hematoxylin staining solution was prepared and dripped onto the tissue sections for 7 min for staining. The differentiation time of acidic ethanol differentiation solution was 5–15 s, after which the tissue sections were rinsed with tap water. After using the blue solution of Masson for 5 min to revert back to the blue colour, the sample was rinsed with tap water and rinsed for 1 min with distilled water. After 7 min, the sample was dyed with fuchsin dye solution and soaked in 0.1% acetic acid solution for 1 min. Phosphomolybdic acid working solution was added, and the sample was dyed for 2 min. Then, the sample was soaked for 1 min in 0.1% acetic acid solution. After 2 min of staining with aniline blue dye, the sample was soaked in 0.1% acetic acid solution for 1 min. The following steps are the same as those for HE staining.

### 2.5 Sirius red

First, Weigert iron hematoxylin staining solution was used to stain the sample for 10–20 min, and then, the sample was rinsed with tap and distilled water. After the distilled water has evaporated, the sample was stained with Sirius red staining solution for 1 h. Finally, the surface dye solution of the slice was removed by rinsing with tap water. The following steps are the same as for HE staining.

### 2.6 Detection of alanine aminotransferase and aspartate aminotransferase

Glutamic oxaloacetic transaminase (AST) and glutamic pyruvic transaminase (ALT) are important indexes in the detection of liver function. Liver tissue (25 mg) was added to 500 μl of RIPA lysate, and centrifugation was performed at 4℃ for 10 min to obtain the supernatant. According to the instructions of the experimental kit (Beyotime, Beijing, China), the absorbance values of each group were detected by multi‐function enzyme labelling instrument.

### 2.7 Determination of hydroxyproline content

First, the ampoule containing liver tissue was placed in an oven and dried at 120°C for 16 h. Then, 3 ml of hydrochloric acid (6 mol/L) was added and hydrolysed at 120°C for 6 h. After cooling to room temperature, the sample was filtered with a 0.45 μM filter membrane and then adjusted to pH 7.2–7.4, and finally, the volume was fixed to 10 ml with pure water. The content of hydroxyproline was detected by chloramine T method.

### 2.8 Quantitative real‐time PCR

Total RNA samples were extracted from cells and tissues by Trizol method. The prepared reverse transcription system was placed in a metal bath at 42℃ and reacted for 15 min. To remove the genome and complete the reverse transcription reaction, the enzyme was completely inactivated at 95℃ for 3 min. Then, the qRT‐PCR reaction system was prepared according to the instructions of Hieff UNICON Power qPCR SYBR Green Master Mix (Yeasen Biotech Co., Ltd.). Primer information is shown in the Table S1.

### 2.9 Western blot

Cell lysate (RIPA: Cocktail = 100:1) was used to extract cell and tissue protein samples. When extracting phosphorylated protein samples, 100× protease inhibitors were added to the cell lysate and diluted to 1×. BCA working solution was used to detect protein concentration. According to the experimental requirements, different concentrations of SDS‐polyacrylamide gel were prepared to separate the proteins. The protein was then transferred to the PVDF membrane. The PVDF membrane was then transferred to 5% skimmed milk powder solution and sealed at room temperature for 1 h. Then, the primary antibody was incubated according to the desired protein molecular weight and shaken overnight at 4℃. After cleaning the PVDF film thrice, it was incubated with the second antibody at room temperature for 2 h and finally detected by chemiluminescence instrument.

### 2.10 MTT assay

The cells were diluted into a cell suspension of 5 × 10^4^/ml and then added to a 96‐well plate and placed in the incubator. On the second day, the corresponding drugs were added to the 96‐well plate, and the zeroing group was set up and cultured for 24 h. Without light, the MTT solution of 5 mg/ml was added to the 96‐well plate; 15 μl was added to each well and incubated in the incubator for 4 h. The liquid from 96 holes was extracted by vacuum pump, and then, DMSO is added to dissolve formazan. The 96‐well plate was placed into the enzyme labelling instrument, and the absorbance value was detected at 570 nm.

### 2.11 Immunofluorescence analysis

The round coverslips were sterilized on the alcohol flame and hung at room temperature. Then, they were placed onto a 24‐well plate and inoculated with a cell density of 5 × 10^4^/ml. After the cells were cultured overnight, they were fixed with 4% paraformaldehyde and permeabilized with 0.2% Triton X‐100. Subsequently, 5% BSA solution was added to each hole for 30 min for sealing. Then, an antibody was added and incubated overnight at 4 ℃ in a wet box. The second antibody was added to the cell round coverslips the next day, and after 1.5 h of incubation without light, it was washed thrice. Finally, the sealed tablet containing DAPI was added. The confocal microscope (LSM 800 with Airyscan, Germany) was used to take pictures.

### 2.12 Flow cytometry apoptosis

Cells were collected as described above. Then, 300 μl Annexin V‐FITC binding solution, 10 ul Annexin V‐FITC, and 10 ul PI were added. The mixture was incubated at room temperature and in the dark for 30 min. Finally, cell apoptosis was detected by flow cytometry (FACSAria III, BD, USA).

### 2.13 Double luciferase report

When the cell growth density was approximately 80%, the cells were added to the 96‐well plate. Luciferase plasmid (Luc‐PI3K‐WT and Luc‐PI3K‐Mut), Lipo8000^™^, and Opti‐MEM culture medium were transfected into cells after overnight culture. Then, the cell lysate was extracted and the luciferase activity was detected by using a multifunctional enzyme label analyser (Thermo Fisher Scientific) according to the instructions (Yeasen Biotech Co., Ltd.).

### 2.14 Statistical analysis

All data were analysed statistically using GraphPad software. All experiments were repeated thrice, and measurement data were expressed as mean ± standard deviation (SD). *T*‐test was used for comparison between two groups, and analysis of variance was used for comparison among multiple groups. *p *< 0.05 indicated that the result was statistically significant.

## 3 RESULT

### 3.1 Idelalisib alleviates liver fibrosis in mice induced by CCl_4_

**FIGURE 1:**
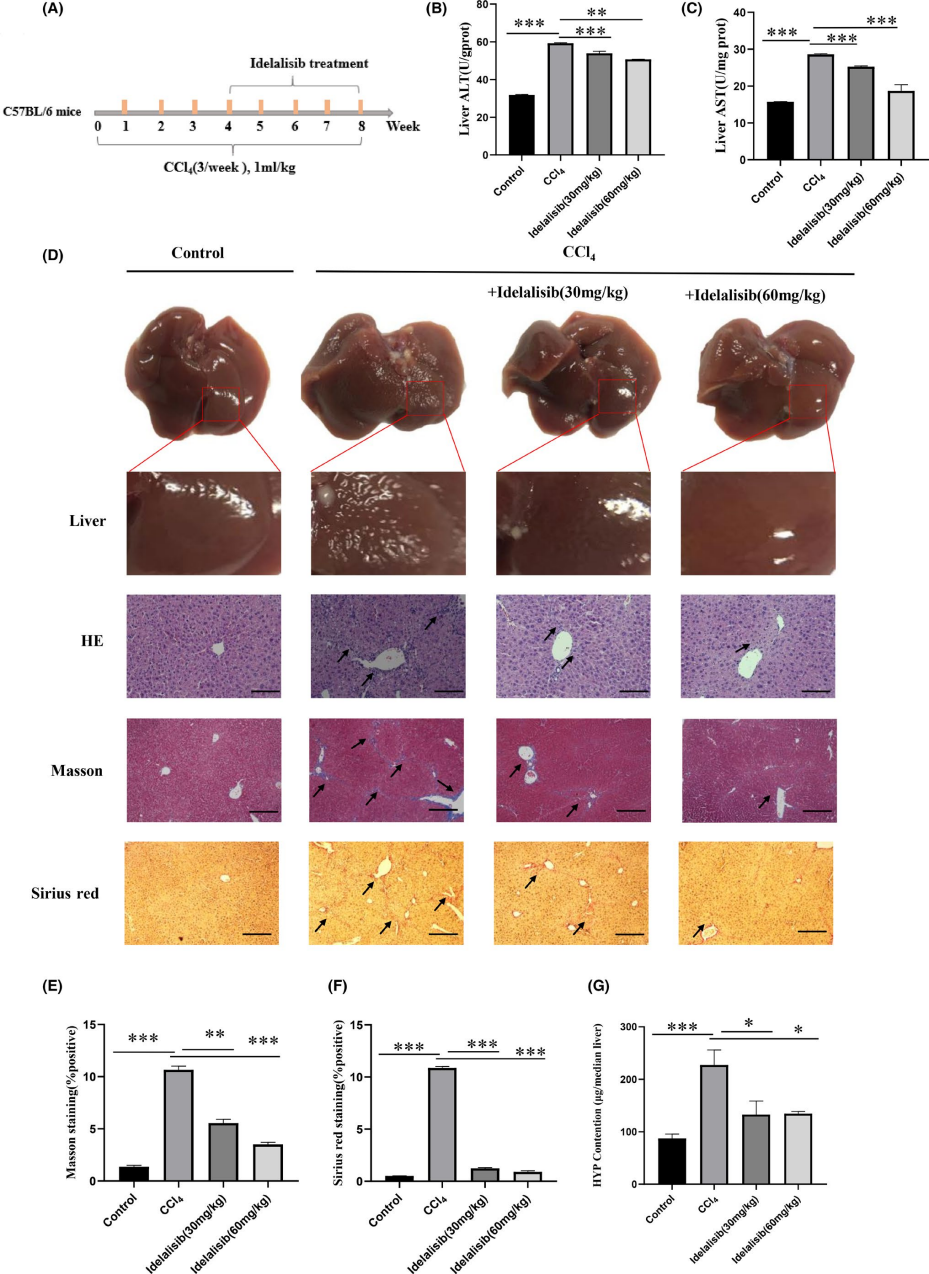
Idelalisib alleviates liver fibrosis induced by CCl_4_ in mice. (A) Time axis of mouse liver fibrosis model induced by CCl_4_. (B) Determination of ALT concentration in liver tissue. (C) Determination of AST concentration in liver tissue. (D–F) Apparent morphology, HE staining, Masson staining, and Sirius red staining results of liver tissue from each group. All results are magnified to 200× field of view. (G) Determination of hydroxyproline in the liver tissue of mice. *p < 0.05, **p < 0.01, ***p < 0.001. *n* = 10

The model was made by intraperitoneal injection of CCl_4_ solution (Figure 1A). Weight analysis showed that compared with the CCl_4_ model group, the weight growth rates of the normal and Idelalisib treatment groups were higher, and growth was faster, thereby indicating that the model group might have liver injury (Figure S1). AST and ALT were the most important indexes to detect liver function injury. Compared with the control group, ALT and AST levels in liver tissue were significantly increased in the CCl_4_ group, whereas ALT and AST levels were significantly decreased in the Idelalisib group (Figure 1B, C). The surface of the liver tissue in the model group was rough, whereas the surface of liver tissue in the Idelalisib group was significantly improved. Similarly, HE staining showed a higher inflammatory infiltration in the model group, Masson staining and Sirius red staining showed a significant increase in collagen fibrous deposition in the model group, whereas these indicators were significantly improved in the Idelalisib group (Figure 1D–F). Further detection of the content of hydroxyproline in liver tissue showed that the content of collagen in CCl_4_ model group significantly increased, and the primary collagen production and deposition in liver tissue significantly improved after treatment with Idelalisib (Figure 1G).

### 3.2 Idelalisib reduces CCl_4_‐induced extracellular matrix deposition

**FIGURE 2:**
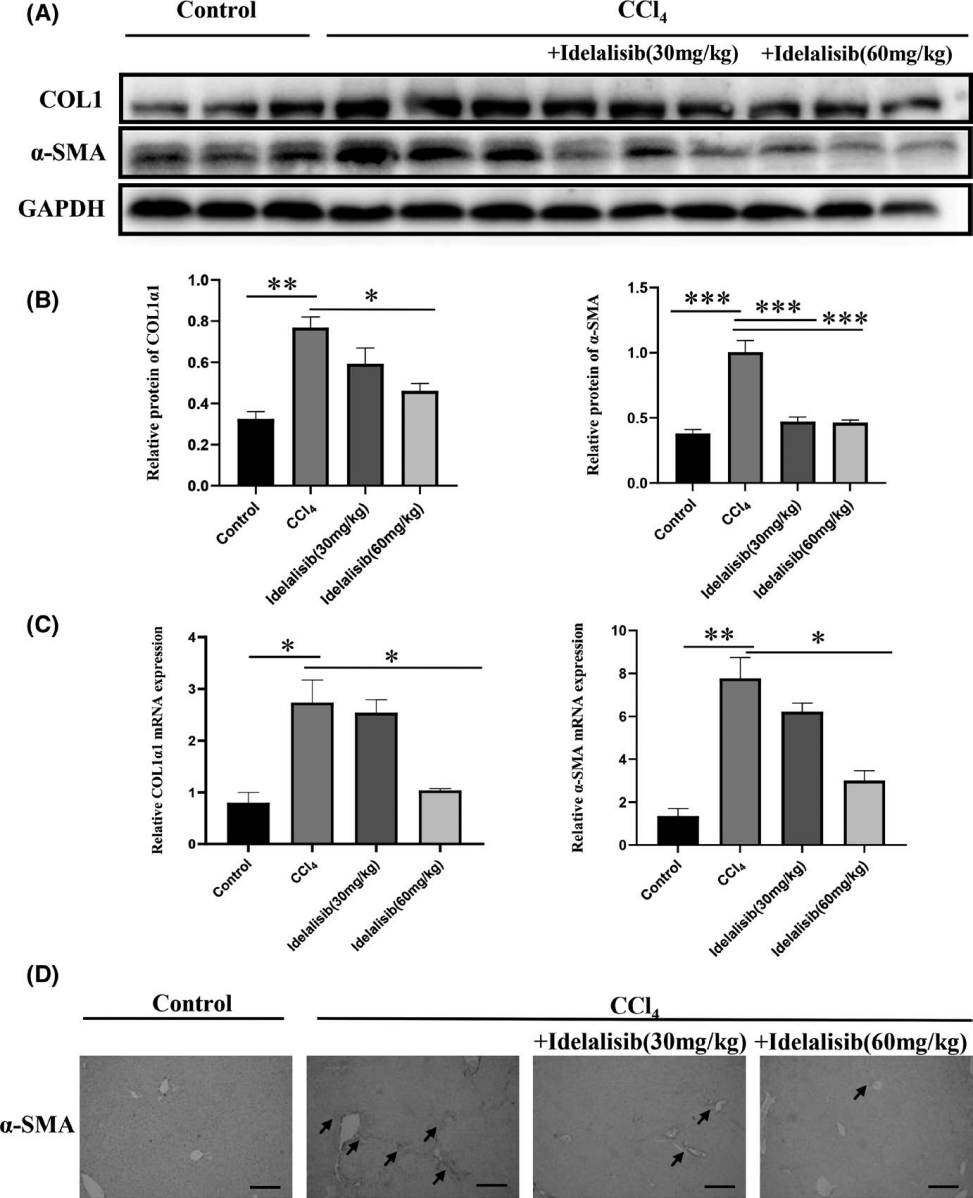
Idelalisib reduces CCl_4_‐induced extracellular matrix deposition. (A and B) Detection of COL1 and α‐SMA protein content in liver tissue by Western blot. (C) Detection of COL1 and α‐SMA gene expressions in vivo by real‐time fluorescence quantitative PCR. (D) Immunohistochemical detection of α‐SMA protein content. All results are magnified to 200× field of view. *p < 0.05, **p < 0.01, ***p < 0.001. *n* = 3

Activated HSCs can secrete α‐SMA, migrate to tissue injury and repair, and secrete a large amount of COL1 and COL1II, thereby increasing ECM, which eventually leads to liver fibrosis. To verify whether Idelalisib can reduce the deposition of extracellular matrix induced by CCl_4_, total protein and total RNA were extracted from liver tissue. The protein and gene expression levels of α‐SMA and COL1 were detected by Western blot analysis and real‐time fluorescence quantitative PCR. The protein and gene expression levels of α‐SMA and COL1 in the CCl_4_ model group were significantly higher than those in the normal group, indicating that CCl_4_ could lead to the accumulation of extracellular matrix in mice liver. The protein and gene levels could significantly reduce the expressions of α‐SMA and COL1 after Idelalisib treatment (Figure 2A–C). At the same time, immunohistochemical staining showed that the expression of α‐SMA protein in the model group was significantly higher than that in the normal group, but Idelalisib could reduce the production of α‐SMA (Figure 2D). The above results confirmed that Idelalisib inhibited the deposition of extracellular matrix and played an anti‐fibre effect.

### 3.3 Idelalisib inhibits HSC activation induced by TGF‐β

Next, we designed 12 concentrations (0–256 μM) to detect the effects of Idelalisib on the viability of LO2 and LX2 cells. When the concentration of Idelalisib was less than or equal to 128 μM, it had no significant effect on the viability of LX2 and LO2 cells (Figure S2Aand Figure S2B). Finally, we chose three concentrations, namely 2.5, 5, and 10 μM for the follow‐up experimental study.

**FIGURE 3:**
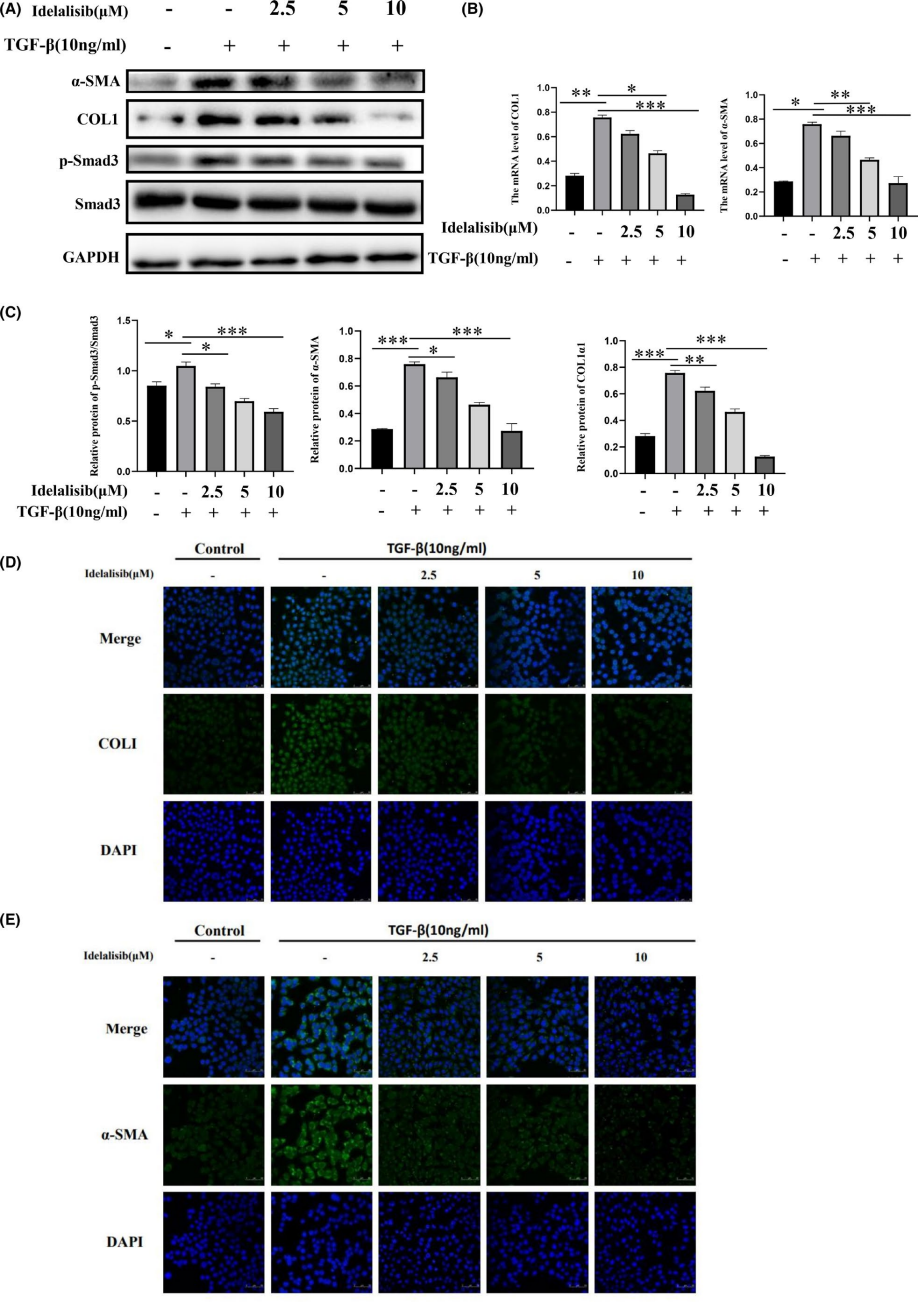
Idelalisib inhibits HSC activation induced by TGF‐β. (A) Protein expressions of p‐Smad3, Smad3, COL1, and α‐SMA in LX2 cells were detected by Western blot. (B) Changes of α‐SMA and COL1 mRNA levels in LX2 were detected by real‐time fluorescence quantitative PCR. (C) Levels of p‐Smad3 COL1 and α‐SMA in Figure 3a were quantitatively analysed using GAPDH as internal reference. (D and E) Immunofluorescence detection of α‐SMA and COL1 protein content in LX2 cells. *p < 0.05, **p < 0.01, ***p < 0.001. *n* = 3

The activation of HSCs is very important in the development of liver fibrosis. Studies have confirmed that TGF‐β is a very important fibrogenic factor. Thus, we chose TGF‐β to stimulate LX2 cells to evaluate the anti‐fibrotic effect of Idelalisib in vitro. Western blot results showed that Idelalisib significantly inhibited the expressions of α‐SMA, COL1, and p‐Smad3/Smad3 in TGF‐β‐stimulated HSC cells (Figure 3A, B). Similarly, real‐time fluorescence quantitative PCR showed that Idelalisib could significantly inhibit the mRNA levels of α‐SMA and COL1 in TGF‐β‐stimulated HSC cells (Figure 3c). Next, we used immunofluorescence to detect the effect of TGF‐β stimulation on the markers of HSC activation, α‐SMA, and COL1 protein. The fluorescence intensity of α‐SMA and COL1 protein increased significantly after TGF‐β stimulation, but the fluorescence intensity of α‐SMA and COL1 significantly decreased in Idelalisib group (Figure 3D, E). In summary, Idelalisib inhibits HSC activation through TGF‐β/Smad3 pathway.

### 3.4 Idelalisib regulates PI3K/AKT/FOXO3 signal pathway to promote apoptosis of activated HSCs

**FIGURE 4:**
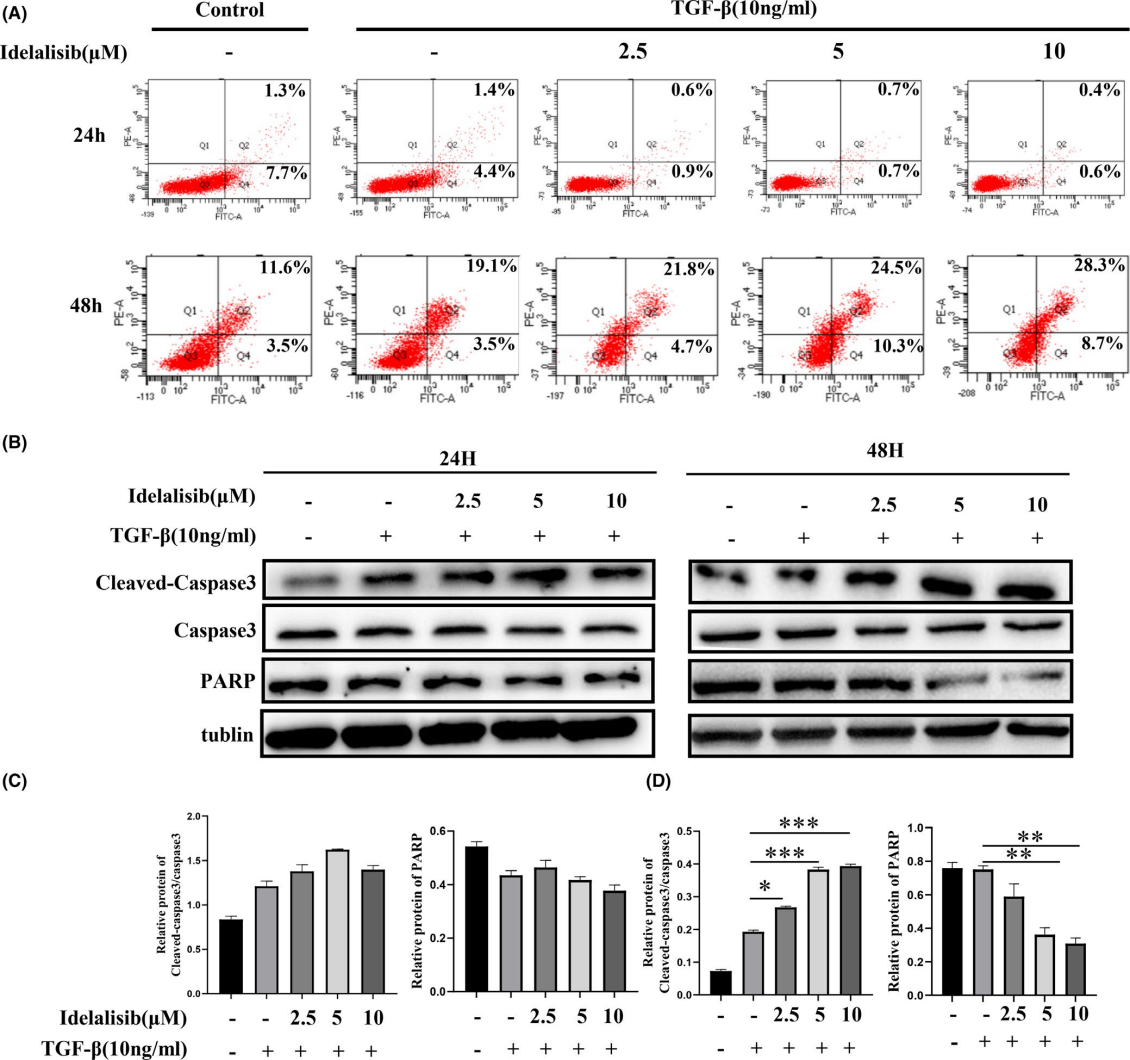
Idelalisib promotes apoptosis of activated HSCs. (A) The effect of Idelalisib on TGF‐β‐induced apoptosis of LX2 cells was detected by flow cytometry. (B–D) Western blot analysis was used to detect the effects of Idelalisib for 24 and 48 h on the expression of cleaved caspase‐3, caspase‐3, and PARP proteins. *p < 0.05, **p < 0.01, ***p < 0.001. *n* = 3

**FIGURE 5:**
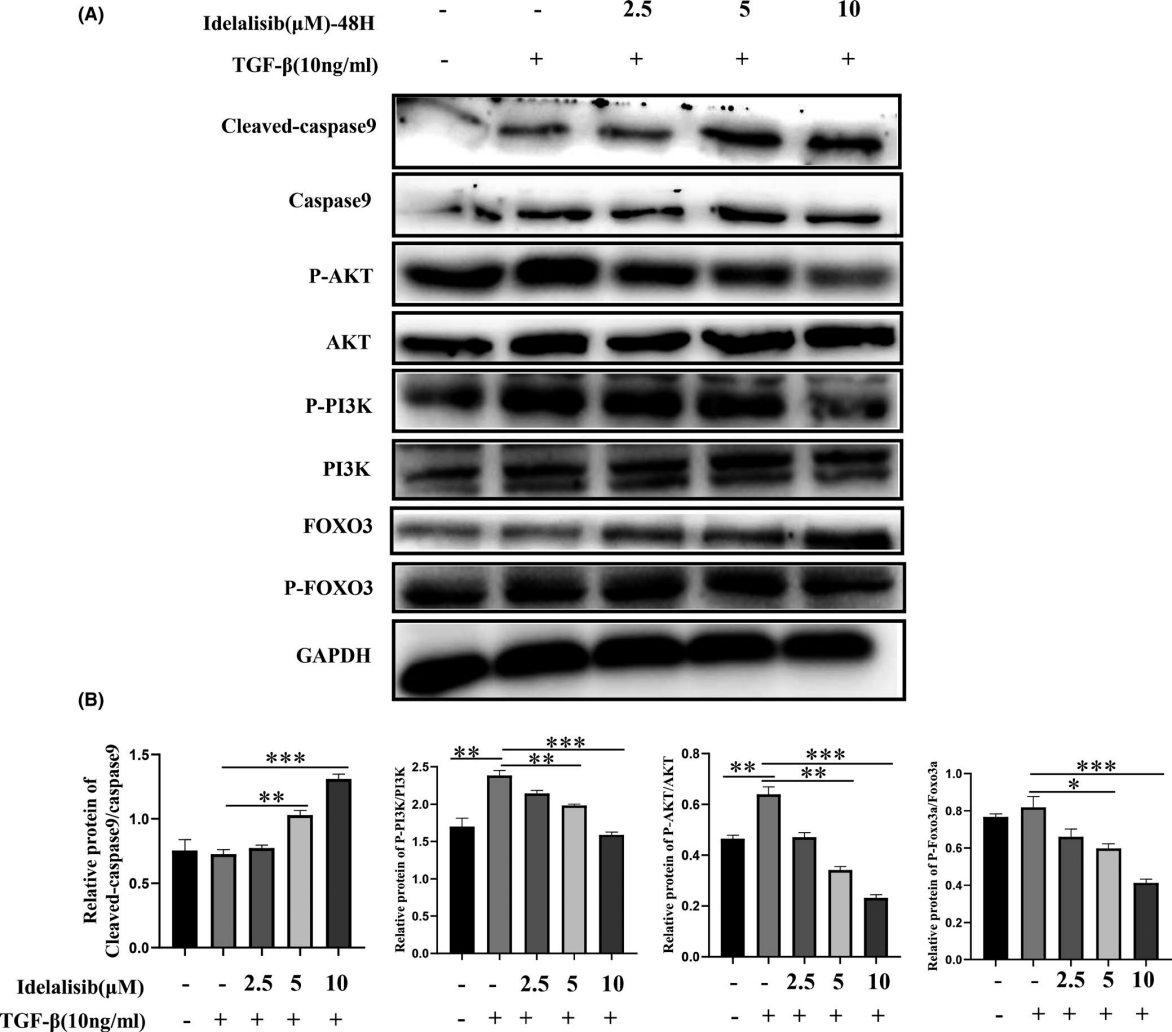
Idelalisib regulates PI3K/AKT/FOXO3 signal pathway to promote apoptosis of activated HSCs. (A and B) Western blot analysis was used to detect the changes of protein expression of cleaved‐caspase‐9, caspase‐9, PI3K/P‐PI3K, AKT/PAKT, and P‐FOXO3/FOXO3 after 48 h of treatment with Adelaris. *p < 0.05, **p < 0.01, ***p < 0.001. *n* = 3

The elimination of activated HSCs can reverse hepatic fibrosis. Then, we detected the effect of Idelalisib on the viability of activated HSCs cells by MTT method. The activity of activated HSCs was not affected when the drug was administered for 24 h. However, the activity of cells was affected when the drug was administered for 48 h at a concentration of 4–32 μM. When the concentration was greater than 32 μM, the growth activity of cells was half or less than that of the normal group (Figure S3A and Figure S3A). Flow cytometry showed that no apoptosis occurred in the activated HSCs after 24 h of treatment with Idelalisib. When we extended the treatment time to 48 h, the activated HSCs showed obvious apoptosis in a gradient‐dependent manner (Figure 4A). The results of Western blot showed that no significant difference was found in the expressions of apoptotic protein cleaved‐caspase‐3 and PARP between the treatment group and the TGF‐β stimulation group for 24 h. However, caspase‐3 protein expression increased in a gradient‐dependent manner when the drug was treated for 48 h, and the expression of PARP decreased significantly, thus inducing apoptosis (Figure 4B–D). Further studies showed that treatment of activated HSCs with Idelalisib for 48 h decreased the expression of p‐PI3K and p‐AKT proteins, leading to the dephosphorylation of FOXO3 (Figure 5A, B). Idelalisib may promote the apoptosis of activated HSC cells by inhibiting the PI3K/AKT/FOXO3 signal pathway.

### 3.5 Overexpression/inhibition of miR‐124‐3p attenuates/enhances PI3K and its phosphorylation

**FIGURE 6:**
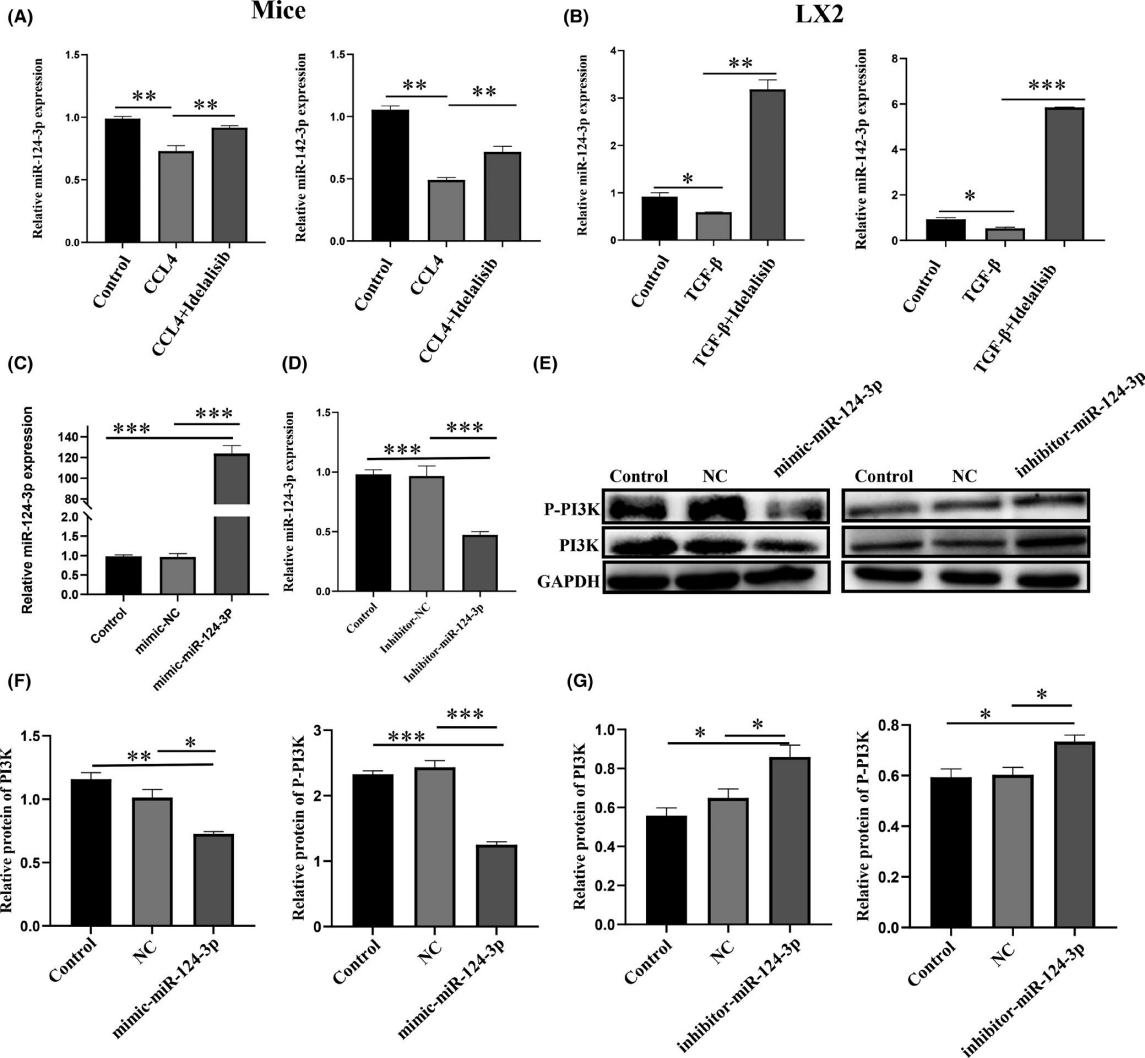
Overexpression/inhibition of miR‐124‐3p attenuates/enhances PI3K and its phosphorylation. (A and B) The changes of miR‐124‐3p and miR‐142‐3p gene expression in liver fibrosis mice and TGF‐β stimulated LX2 cells were detected by real‐time fluorescence quantitative PCR. (C and D) The effect of miR‐124‐3p inhibitor/mimic on miR‐124‐3p gene level was detected by real‐time fluorescence quantitative PCR. (E–G)Western blot analysis was used to detect the effect of inhibitor/mimic on the expression of P‐PI3K and PI3K proteins. *p < 0.05, **p < 0.01, ***p < 0.001. *n* = 3

Many liver diseases are associated with abnormal expression of miRNAs, and the development of liver fibrosis may also involve the extensive participation of miRNA. Many studies have found that miRNA can act as a negative regulator of PI3K to inhibit gene expression. We hypothesized that Idelalisib inhibits PI3K from promoting the apoptosis of activated HSCs by regulating miRNA. First, the targeted miRNAs of PI3K were detected through Diana, miRanda, and miRBridge databases and intersected to obtain three miRNAs. Then, these three miRNAs were intersected with miRNAs related to CCl_4_‐induced liver fibrosis reported in recent years, and hsa‐miR‐124‐3p and hsa‐142‐3p were finally determined (Figure S4A and Figure S4B). Subsequently, we found that Idelalisib could up‐regulate miR‐124‐3p and miR‐142‐3p both in vivo and in vitro (Figure 6A, B). Since miR‐142‐3p has no binding sequence in the 3 ‘untranslated region of PI3K gene, we only conducted subsequent studies on miR‐124‐3p (Figure S4C). We proved that miR‐124‐3p mimic and inhibitor could overexpress and silence miR‐124‐3p and could silence or upregulate p‐PI3K and PI3K (Figure 6C–G).

### 3.6 Idelalisib exerts pro‐apoptotic effects by inhibiting the PI3K/Akt pathway through miR‐124‐3p

**FIGURE 7:**
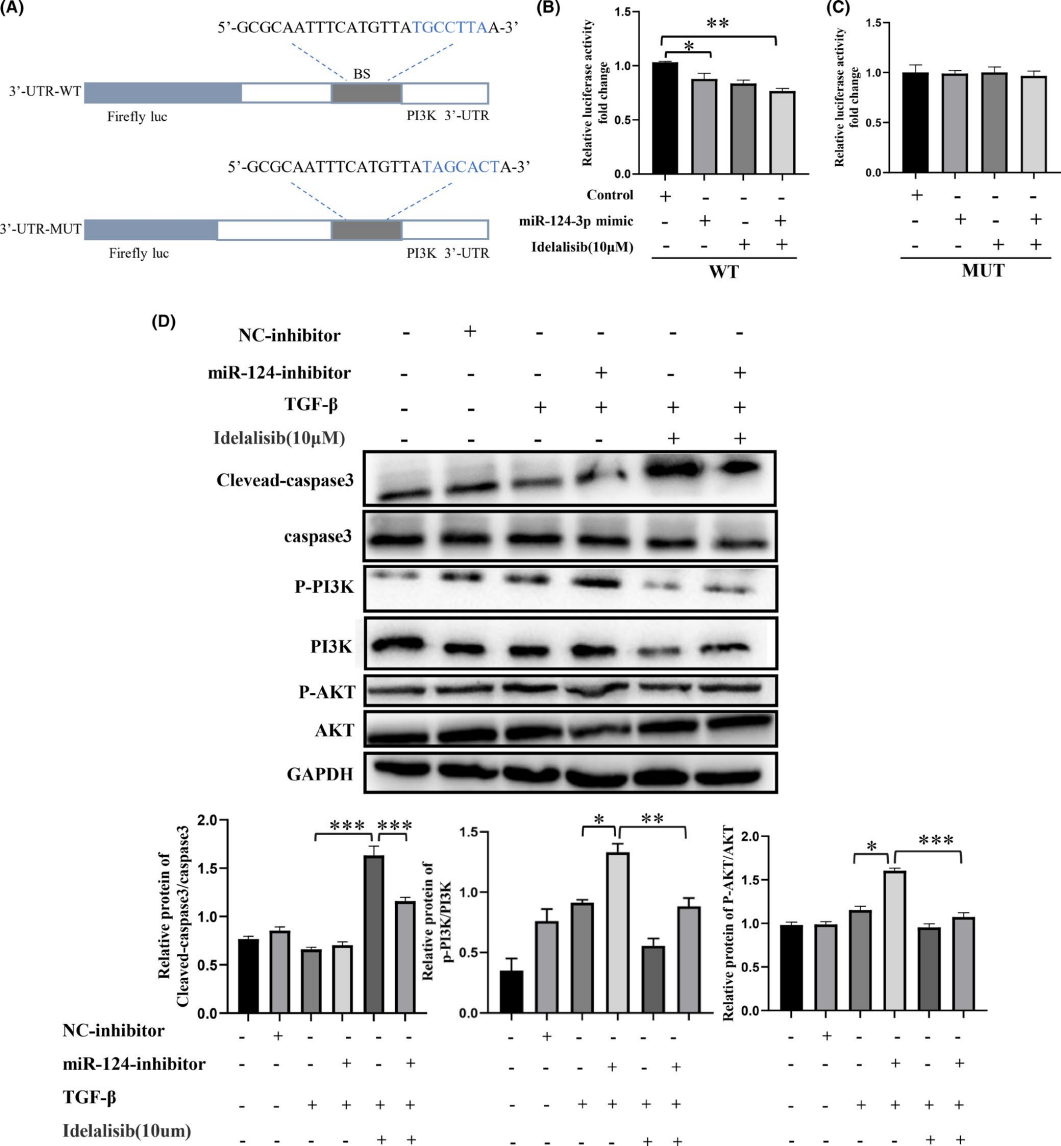
Idelalisib exerts pro‐apoptotic effects by inhibiting the PI3K/Akt pathway through miR‐124‐3p. (A) Dual‐luciferase gene reporter plasmid containing P13K wild‐type and mutant sequences. (B and C) Luciferase was used to detect the effect of miR‐124‐3P mimic and Idelalisib on fluorescence intensity. (D) Western blot analysis was used to detect the effects of miR‐124‐3p inhibitor and Idelalisib on PI3K/Akt signalling pathway and apoptotic proteins. *p < 0.05, **p < 0.01, ***p < 0.001. *n* = s3

To further verify whether Idelalisib inhibits PI3K by miR‐124‐3p, we constructed luciferase‐PI3K‐WT and luciferase‐PI3K‐MUT plasmids (Figure 7A). The results of the dual luciferase report showed that in the luciferase‐PI3K‐WT report, Idelalisib could significantly inhibit luciferase activity, whereas in the luciferase‐PI3K‐MUT report, no significant effect was found (Figure 7B, C). Finally, miR‐124‐inhibitor was used to detect the mechanism of Idelalisib regulation of PI3K, and miR‐124‐3p was found to inhibit the expression of cleaved caspase‐3 in activated HSC cells and activate the PI3K pathway. However, these effects were reversed by Idelalisib (Figure 7D). In summary, these results confirmed that Idelalisib can reverse the effect of miR‐124‐3p inhibitor.

## 4 DISCUSSION

In the whole process of liver fibrosis, the continuous activation of HSCs is the most critical event that leads to liver fibrosis.^17^ HSC activation can be divided into two stages: initial activation and permanent activation. If the factors that induce fibrosis disappear, the activated HSC may develop to stage 3: regression.^18^ In the initial stage, HSC receives molecular signals from neighbouring cells that secrete fibroblast and growth factors, enhancing the transformation of HSC into myofibroblasts and secreting fibrogenic factors.^18^ After activation, HSC proliferates and secretes a large amount of ECM protein, which leads to the accumulation of ECM and scar formation. CCl_4_‐induced liver injury in mice can be further developed into a fibrosis model, which is a classical early liver fibrosis model that can well simulate the pathological characteristics of human liver fibrosis in the S2–S4 stages.^19^, ^20^ In the present study, we demonstrated that Idelalisib alleviated CCl_4_‐induced liver fibrosis in mice, as shown by reducing ALT, AST, and tissue collagen deposition.

With the in‐depth study of the cellular and molecular mechanism of hepatic fibrosis, the abnormal activation of HSCs is usually accompanied by the activation of TGF‐β/Smad signal pathway.^21^, ^22^ Zhao et al.^23^ found that ferulic acid alleviates liver fibrosis by inhibiting the TGF‐β/Smad signalling pathway. Another study also demonstrated that methyl ferulate alleviated liver fibrosis and HSC activation through the TGF‐β1/Smad pathway.^24^ In the present study, we stimulated LX2 cells with TGF‐β as the activation model of HSCs, and in vitro studies showed that Idelalisib inhibited the activation of TGF‐β/Smad3 signalling pathway and significantly reduced the expression of COL1 and α‐SMA gene and protein levels, thereby inhibiting the activation of HSCs.

Abnormal activation of HSCs was the most critical link that leads to liver fibrosis. Inhibition of HSC activation can prevent or slow down the development of liver fibrosis, and removal of activated HSCs can reverse liver fibrosis.^4^, ^25^, ^26^ Meng et al. found that carvedilol can significantly reduce the number of activated HSCs and induce their apoptosis, thereby alleviating hepatic fibrosis.^27^ Alberto et al. found that rilpivirine can selectively induce apoptosis of activated HSCs and alleviate liver fibrosis through the activation of signal transducer and the activator of transcription‐1.^28^ We used LX2 cells stimulated by TGF‐β as activated HSCs. The cell activity was significantly inhibited, and apoptosis occurred in activated HSCs after treatment with Idelalisib for 48 h. The above studies have shown that Idelalisib can induce apoptosis in HSCs, but its mechanism has not been elucidated. Idelalisib can reportedly inhibit AKT/FOXO3a pathway to promote Bim activation and induce HCC cell apoptosis, thus treating hepatocellular carcinoma.^29^ Idelalisib can improve fibrotic ophthalmopathy by inhibiting PI3K/AKT.^30^ Based on PI3K/AKT, Idelalisib plays an important role in the activation and proliferation of HSCs. No report has mentioned whether Idelalisib inhibits the expression of PI3K/AKT in hepatic fibrosis. By detecting the expression of PI3K/AKT and downstream apoptotic proteins, we found that Idelalisib could promote the apoptosis of activated HSCs induced by downstream apoptotic proteins cleaved‐caspase‐9 and cleaved‐caspase‐3 by inhibiting PI3K/AKT/FOXO3a signal pathway. Surprisingly, Idelalisib (10 uM) could reduce the level of p‐PI3K and inhibit the level of PI3K. We speculated that Idelalisib may regulate PI3K expression through a mechanism, thereby affecting the reduction of PI3K phosphorylation.

MiRNAs do not act on genes directly, but they act on the mRNA transcribed by genes to down‐regulate the expression of target genes, thus affecting protein expression. With the in‐depth study of miRNA, many miRNA were found to be involved in regulating the development of liver fibrosis. The expression of miR‐194 is down‐regulated in human liver fibrosis and activated HSCs. Overexpression of miR‐194 can inhibit targeted protein kinase to reduce the protein levels of α‐SMA and COL1, thereby inhibiting the activation of HSCs and alleviating liver fibrosis.^12^ The miR‐1273g‐3p may exert its anti‐fibrosis effect through the PTEN/AKT signal pathway.^31^ Through database search and literature research, we predicted the upstream miRNA that regulates PI3K. Both in vivo and in vitro experiments showed that the levels of miR‐124‐3p in CCl_4_ model and TGF‐β stimulation groups were significantly lower than that in the normal group, whereas the level of miR‐124‐3p gene was significantly up‐regulated after treatment with Idelalisib. Overexpression or silencing of miR‐124‐3p can significantly inhibit or promote PI3K expression.

Finally, we further proved that Idelalisib can reverse the activation of the miR‐124‐3p inhibitor on the PI3K/AKT pathway, thus promoting apoptosis. Our results demonstrated that Idelalisib inhibited the PI3K/AKT pathway by regulating miR‐124‐3p and thus played a pro‐apoptotic role. In summary, this study found that Idelalisib can alleviate hepatic fibrosis by inhibiting the activation of HSCs and inducing apoptosis. Results discussed the mechanism by which Idelalisib regulates PI3K.

## 5 CONCLUSION AND PROSPECT

As liver fibrosis is affected by many factors, the formation process and mechanism are complex. No clinical drugs for hepatic fibrosis have been developed. In recent years, with the gradual deepening of the research on the molecular pharmacological mechanism of liver fibrosis, many key targets and signal pathways that may be useful in the treatment of liver fibrosis have been found. By investigating the listed drugs with related targets or signal pathways related to liver fibrosis, we can explore new therapeutic drugs from the list. The safety of the drugs on the market is guaranteed to a certain extent, thereby greatly reducing the cost of research and development, shortening the time of drug development, and providing more treatment options for patients with liver fibrosis. The limitation of this study is as follows: the inherent target of Idelalisib was explored, but its other targets in liver fibrosis were not explored. An in‐depth study that involves follow‐up experiments is required to determine whether Idelalisib has an anti‐fibrotic effect in coordination with other targets.

## CONFLICT OF INTEREST

The authors declare no conflicts of interest.

## AUTHOR CONTRIBUTIONS

**Xiaohe Li:** Conceptualization (equal); Data curation (equal). **Hailong Li:** Conceptualization (equal); Data curation (equal); Formal analysis (equal); Project administration (equal). **Shanshan Zhang:** Formal analysis (equal); Investigation (equal); Methodology (equal). **Ruotong Zhang:** Software (equal). **Jinhe Li:** Software (equal); Supervision (equal); Visualization (equal). **Yiying Wei:** Software (equal); Writing‐review & editing (equal). **Fubo Zhang:** Formal analysis (equal); Methodology (equal); Supervision (equal); Writing‐original draft (equal). **Honggang Zhou:** Conceptualization (equal); Data curation (equal); Funding acquisition (equal). **Cheng Yang:** Conceptualization (equal); Funding acquisition (equal).

## Supporting information

Figures S1‐S4

Table S1
