# Supplementary material for: Protective effect of Idelalisib on carbon tetrachloride‐induced liver fibrosis via microRNA‐124‐3P/phosphatidylinositol‐3‐hydroxykinase signalling pathway
Source: J Cell Mol Med. 2021 Nov 7;25(24):11185–97. doi: 10.1111/jcmm.17039 (PMC8650042; doi:10.1111/jcmm.17039)
Supplement: Supplementary file 1 — Figures S1‐S4 [file JCMM-25-11185-s001.pdf]

Fig s1

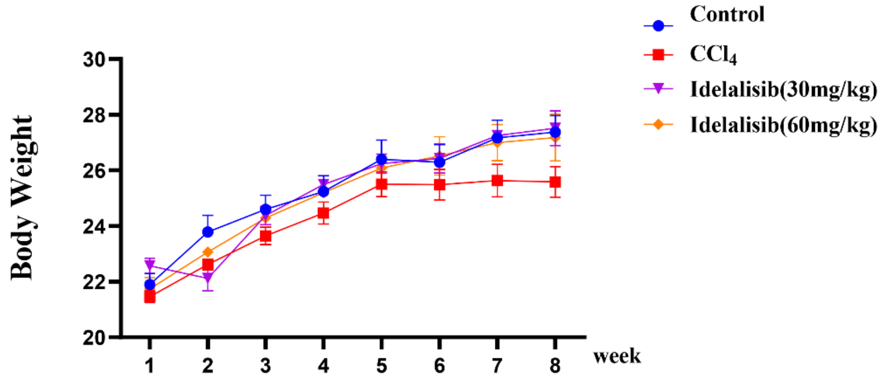

Fig s1. Changes in body weight of mice from 1 to 8 weeks

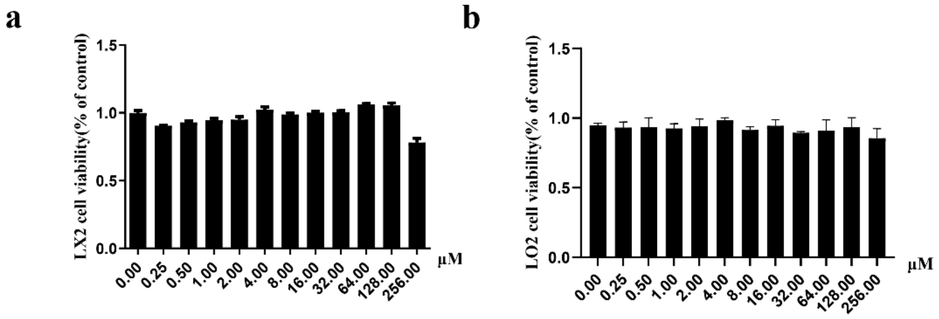

Fig s2 a, b Effect of Idelalisib on the viability of LO2 and LX2 cells.

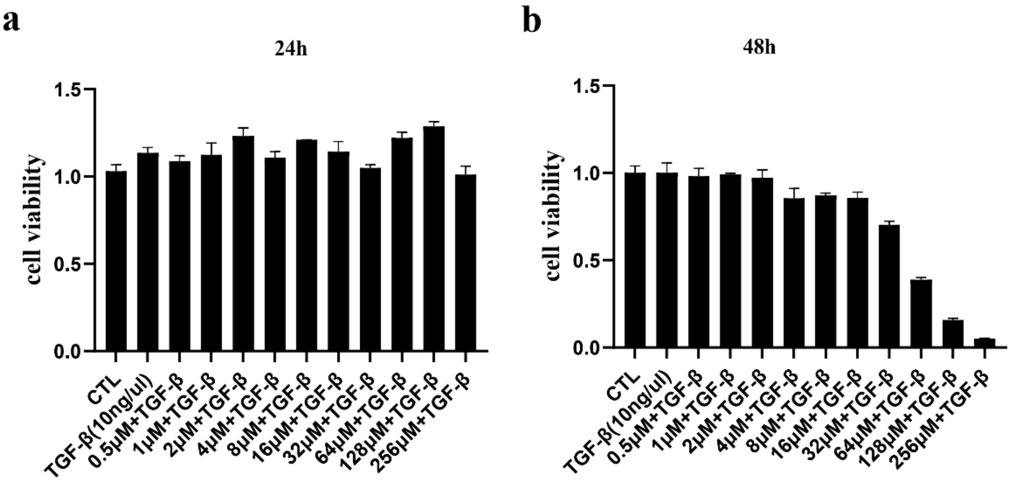

Fig s3 a, b MTT was used to detect the effect of 24 h and 48 h Idelalisib on TGF-β-induced LX2 cell viability.

a

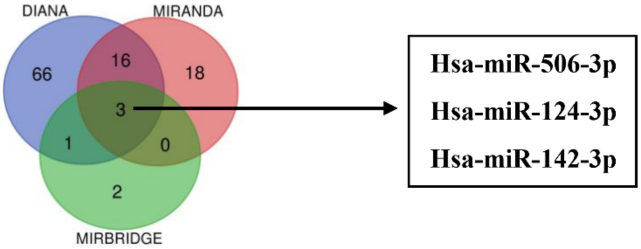

b

Down-regulation of miRNA in CCL4-induced liver fibrosis model

miR-26b-5p	miR-29b-3p	miR-30a	miR-34a-5p	miR-206	miR-98
**miR-124-3p**	miR-145	miR-194	miR-200a	miR-219	miR-378
miR-489-3p	miR-19b	miR-29b	miR-130a-3p	miR-134	**miR-142-3p**
miR-193a-3p	miR-375-3p	miR-455-3p	miR-875-5p		

c

PI3K-wt 3'UTR	5'	GCGCAATTTCATGTTATGCCTTAA
Has-miR-124-3p	3'	AACCGUAAGUGGCGCACGGAAU
P13K-mut 3'UTR	5'	GCGCAATTTCATGTTATAGCACTA

Fig s4 a Diana, MIRANDA and MIRBRIDGE databases were used to analyze the upstream miRNAs that regulate PI3K gene. **b** Downregulated miRNAs in CCl4-induced liver fibrosis models. **c** Part of the gene sequences and binding sites of the wild type and mutant of miR-124-3p and PI3K.
