# Supplementary figures and images for: Protective effect of Idelalisib on carbon tetrachloride‐induced liver fibrosis via microRNA‐124‐3P/phosphatidylinositol‐3‐hydroxykinase signalling pathway
Source: J Cell Mol Med. 2021 Nov 7;25(24):11185–97. doi: 10.1111/jcmm.17039 (PMC8650042; doi:10.1111/jcmm.17039)

Supplementary Table S1: The target gene primer sequence was used in the experiment


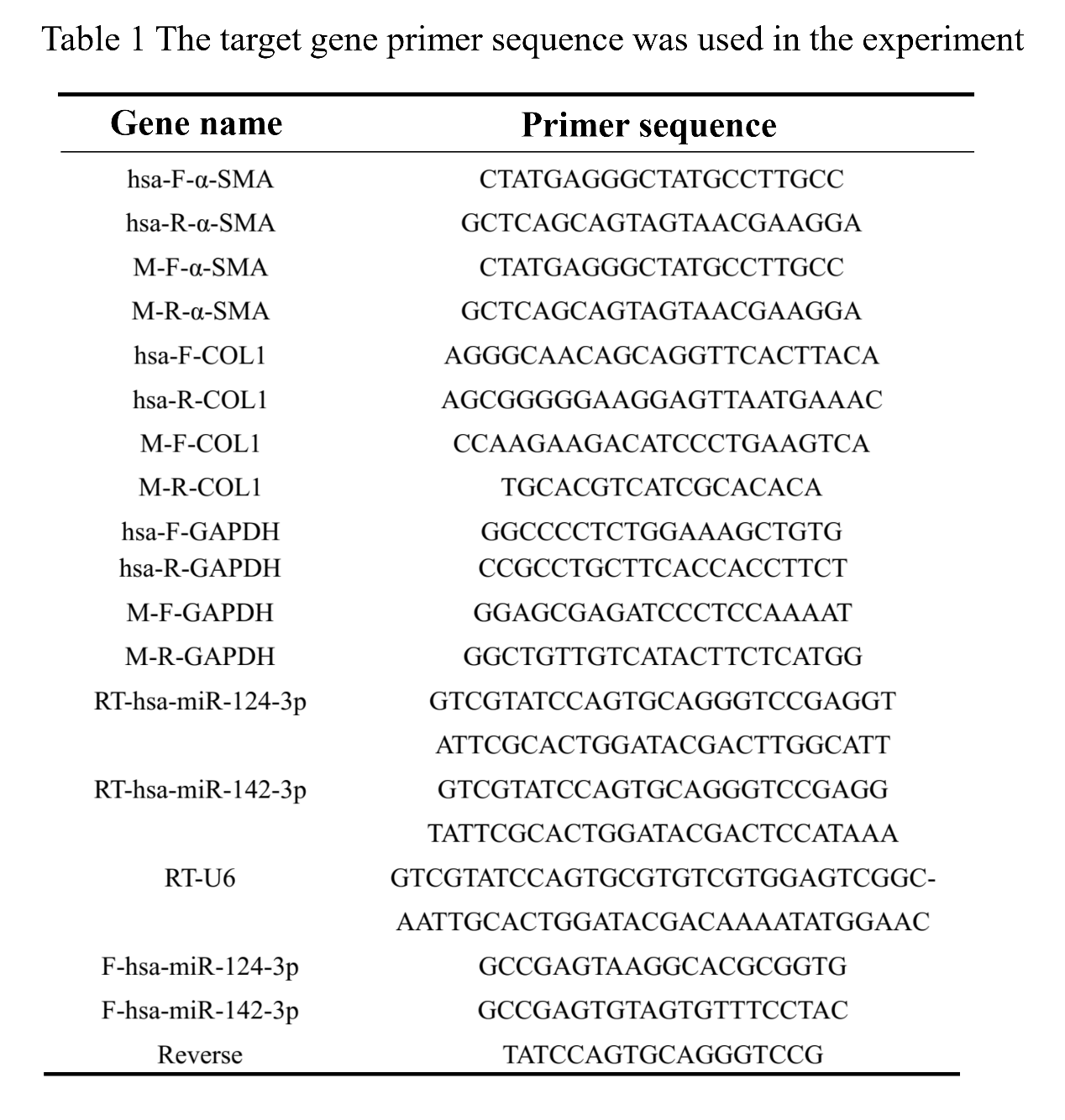

Supplement: Supplementary file 2 — Table S1 [file JCMM-25-11185-s002.docx]
